# PPA Tele-Savvy: Results of an Online Pilot Intervention With Caregivers of Persons With Primary Progressive Aphasia

**DOI:** 10.1093/geroni/igaf122.017

**Published:** 2025-12-31

**Authors:** Darby Morhardt, Lauren Dowden, Kaitlyn Lucca

**Affiliations:** Northwestern University, Evanston, Illinois, United States; Northwestern University, Evanston, Illinois, United States; Northwestern University, Evanston, Illinois, United States

## Abstract

A PPA diagnosis leads to complex psychosocial issues compounded by complicated terminology and uncertain progression and pathology. Dementia caregiver interventions do not match PPA families’ needs for tailored support. Project aims: (1) Identify PPA families’ most pressing challenges and find ways the existing evidence-based caregiver intervention Tele-Savvy should be adapted. (2) Pilot the resulting PPA Tele-Savvy intervention with PPA care partners in 7 weekly 90 minute videoconferences using formative evaluation to address communication, cognitive and behavioral changes, and assist caregivers in achieving competence in the caregiving role. Fourteen spousal caregivers participated in Aim 1 and identified ways Tele-Savvy was helpful and ways it was not. Revisions were incorporated into the resulting PPA Tele-Savvy caregiver intervention. Fifteen spousal caregivers participated in 2 pilot interventions (n = 9, n = 6) involving seven 90-minute weekly videoconference sessions, mindfulness exercises, PPA-specific manual readings, and homework assignments. Pre-post results demonstrated trends in decreased caregiver depression, increased competency, decreased burden, increased PPA knowledge and significant increased positive dyadic interaction. Analysis of video transcriptions and evaluative focus groups revealed: increasing knowledge of PPA, learning new approaches of communication and connection, adjusting expectations, initiating care planning, and assessing self-care. PPA Tele-Savvy demonstrates the feasibility of a tailored PPA caregiver intervention and yields positive results for improving caregiver well-being, competence, knowledge, with positive effects on the dyadic relationship. Further testing in a larger trial is warranted.

